# Efficacy and Safety of Avatrombopag Treatment in Immune Thrombocytopenia Patients in Poland: A Multicenter Study

**DOI:** 10.1055/a-2868-3290

**Published:** 2026-05-13

**Authors:** Michal Witkowski, Jagoda Rytel, Justyna Kozinska, Jolanta Oleksiuk, Weronika Piszczek, Elzbieta Wiater, Jacek Kwiatkowski, Janusz Halka, Radoslaw Bogucki, Wiktoria Ryzewska, Tadeusz Robak

**Affiliations:** 1 Hematology Outpatient Clinic, Copernicus Memorial Hospital, Lodz, lodzkie, Poland; 2 Department of Science and Technology, Foundation for Leukemia Patients, Lodz, Poland; 3 37803 Department of Hematology, Transplantation and Internal Medicine, Medical University of Warsaw, Warsaw, Masovian Voivodeship, Poland; 4 Department of Hematooncology and Bone Marrow Transplantation, Medical University of Lublin, Lublin, Lublin Voivodeship, Poland; 5 Hematology Clinic, University Clinical Hospital in Bialystok, Bialystok, Podlaskie Voivodeship, Poland; 6 Department of Hematology, Rydygier Provincial Polyclinical Hospital, Torun, Poland; 7 Myeloma and Amyloidosis Treatment Center, Department of Hematology and Bone Marrow Transplantation, Nicolaus Copernicus Hospital, Torun, Poland; 8 Hematology and Transplantology Center, Lower Silesian Oncology, Pulmonology and Hematology Center, Wroclaw, Poland; 9 Department of Hematology and BMT, Clinical Hospital of the Ministry of Internal Affairs and Administration with the Warmia-Masuria Oncology Centre, Olsztyn, Poland; 10 Department of General Hematology and Internal Medicine, Copernicus Memorial Hospital, Lodz, Poland; 11 Haematology Clinic, Medical University of Lodz, Lodz, Lodz Voivodeship, Poland

**Keywords:** immune thrombocytopenia, avatrombopag, thrombopoietin receptor agonists, real-world study, treatment response predictors

## Abstract

Immune thrombocytopenia (ITP) is an autoimmune disorder characterized by platelet counts < 100 ×10 ^9^ /L and variable risk of bleeding. Many adults develop chronic disease requiring second-line therapy. Avatrombopag, an oral thrombopoietin receptor agonist (TPO-RA), has demonstrated high efficacy and favorable safety in clinical trials, but real-world data from Poland are lacking.

## Introduction

Immune thrombocytopenia (ITP) is an acquired autoimmune disorder characterized by isolated thrombocytopenia (platelet count < 100 × 10 ^9^ /L) and an increased risk of bleeding. The global incidence of ITP varies by age group, but in adults, it is generally estimated at 1.5 to 2.7 cases per 100,000 individuals per year, while prevalence estimates range more broadly (4–24 per 100,000). Most adults develop a chronic, relapsing form of the disease. 1 The clinical course is heterogeneous: some patients remain asymptomatic, whereas others present with recurrent mucocutaneous bleeding or, less commonly, life-threatening hemorrhage. 2 3 Beyond bleeding risk, ITP imposes a substantial physical and psychological burden. A recent multinational survey showed that living with chronic ITP negatively affects daily activities, travel, sleep, eating habits, exercise, and mental well-being, highlighting the importance of long-term disease control and treatment tolerability. 4

First-line treatment for adult ITP focuses on decreasing platelet destruction and modulating the immune response. Standard first-line therapy consists primarily of corticosteroids, with intravenous immunoglobulin (IVIG) used in patients requiring a rapid platelet increase or presenting with significant bleeding. 5 6 However, these therapies are associated with variable and often transient responses, and relapse is common after tapering or discontinuation. Moreover, long-term corticosteroid use is limited by well-known toxicities. Consequently, many patients require second-line treatments, such as thrombopoietin receptor agonists (TPO-RAs), rituximab, immunosuppressants, danazol, or splenectomy. 5 7 8 9 In refractory cases with limited remaining therapeutic options, vinca alkaloids may also be considered as a last-resort approach, primarily to achieve a short-term platelet increment. 10

TPO-RAs have become a cornerstone of second-line therapy in chronic ITP. By activating the thrombopoietin receptor (c-Mpl) on megakaryocyte progenitors, these agents enhance megakaryocyte proliferation and differentiation, thereby increasing platelet production and addressing a central mechanism of ITP pathophysiology. 11 12 13 Eltrombopag and romiplostim, 2 second-generation TPO-RAs, 14 15 have been shown to be effective and generally well tolerated, and are widely recommended as second-line therapy in adult ITP, irrespective of splenectomy candidacy, particularly in patients with persistent thrombocytopenia or increased bleeding risk. 16 17 18 Nevertheless, both agents have important limitations. Eltrombopag is sometimes associated with elevations in alanine aminotransferase and bilirubin and carries a boxed warning for severe, potentially life-threatening hepatotoxicity, necessitating regular liver function monitoring. It also requires strict dietary restrictions and separation from certain foods and polyvalent cations, which may negatively impact adherence. 4 19 Romiplostim, administered as weekly subcutaneous injections, is less convenient for patients, who prefer oral administration. 17 20 These limitations underline an unmet need for orally administered TPO-RAs with improved safety, more convenient dosing, and fewer practical constraints.

Avatrombopag is a small-molecule, second-generation oral TPO-RA that mimics the biological effects of thrombopoietin in vitro and in vivo, thereby promoting megakaryocyte differentiation and platelet production. 21 22 Unlike eltrombopag, avatrombopag can be administered with food and has not been associated with clinically significant hepatotoxicity in clinical studies, avoiding both dietary restrictions and the safety concerns related to hepatotoxicity and parenteral administration seen with other TPO-RAs. 23 24 25 In phase 2 trials, oral avatrombopag increased platelet counts and was more effective than placebo in achieving platelet responses at day 28, with most adverse events (AEs) reported as mild to moderate in severity. 16

The pivotal phase 3 multicenter, randomized, double-blind, placebo-controlled study by Jurczak et al evaluated avatrombopag 20 to 40 mg once daily in adults with chronic ITP and baseline platelet counts < 30 × 10 ^9^ /L. Avatrombopag was superior to placebo in the cumulative number of weeks with platelet counts ≥ 50 × 10 ^9^ /L without rescue therapy over 6 months (median 12.4 weeks vs. 0.0 weeks; *p* < 0.0001), achieved a platelet response in 65.6% of patients by day 8, and significantly increased the rate of durable platelet response. 26 The safety profile was consistent with earlier phase 2 data, with headache and contusion being the most common AEs, and no new safety signals were identified. A subsequent systematic literature review and network meta-analysis confirmed that avatrombopag has a high probability of achieving durable platelet responses and reducing bleeding events compared with placebo and other TPO-RAs, 27 while real-world studies have shown effectiveness in patients intolerant or unresponsive to other TPO-RAs. 18 A recent meta-analysis did not demonstrate a significant increase in thrombotic risk with TPO-RAs, including avatrombopag. 28 However, other studies have suggested a potential association, and the thrombotic risk of TPO receptor agonists in patients with ITP remains a matter of ongoing debate. 29 30

The phase 3 trial by Jurczak et al included centers in Poland and provided robust randomized evidence of avatrombopag efficacy and safety in chronic ITP. 26 However, randomized clinical trial populations are highly selected and may not fully reflect real-world treatment patterns, comorbidities, or the characteristics of heavily pretreated patients encountered in routine practice. In addition, while several countries have established ITP registries and generated real-world evidence on TPO-RAs, there are currently no national data describing avatrombopag use in Poland.

The aim of this study was to evaluate the real-world efficacy and safety of avatrombopag in Polish adults with primary ITP, as well as to assess response durability, treatment-free remission (TFR) rates, and clinical predictors of treatment response and loss of response. By providing real-world data from eight tertiary centers across Poland, this multicenter study seeks to complement the phase 3 findings and clarify the role of avatrombopag in the everyday management of chronic ITP in Poland.

## Materials and Methods

### Study Design

To investigate the real-world use of avatrombopag in Poland, we conducted a retrospective multicenter analysis of all consecutive patients with primary ITP who initiated treatment with avatrombopag across eight hematology centers in Poland. The study included patients treated between 2021 and 2025. Clinical and laboratory data were retrieved from electronic medical records according to a predefined extraction protocol.

The diagnosis of primary ITP was established based on a comprehensive clinical and laboratory assessment, following the criteria of the International Working Group and the 2019 American Society of Hematology guidelines, 6 which form the basis of the national Polish ITP recommendations. Adult patients (≥18 years) were eligible if they presented with a platelet count ≤ 30 × 10 ^9^ /L, or <50 × 10 ^9^ /L in the presence of bleeding symptoms or when receiving anticoagulants or in the context of planned invasive procedures. All patients had previously received at least one line of ITP therapy in accordance with the requirements of the Polish drug reimbursement program. Patients with prior exposure to avatrombopag were excluded. Additional exclusion criteria included secondary causes of thrombocytopenia, inherited thrombocytopenias, incomplete medical records, or an uncertain diagnosis.

Clinical and laboratory information was collected at the initiation of avatrombopag. These data included age, autoimmune comorbidities, viral serology results, findings from bone marrow examination, history of splenectomy or other relevant procedures, number and type of previous treatment lines, and prior therapeutic exposures. We also recorded concomitant ITP medications, bleeding episodes, thrombotic events, and laboratory parameters such as complete blood count, platelet production indices, and basic biochemical tests. Follow-up clinical and laboratory assessments were performed 1, 3, 6, and 12 months after the start of avatrombopag therapy, with complete blood count evaluated weekly during initial dose adjustment and liver function tests performed monthly.

### Treatment

Avatrombopag was administered orally once daily. The standard starting dose was 20 mg/day, with subsequent dose adjustments individualized according to platelet counts, clinical response, and treatment tolerability. In routine clinical practice, weekly doses typically ranged from 10 to 280 mg. Although 20 mg represents the minimum dose specified in the summary of product characteristics (SmPC), a 10 mg weekly dose was used in selected cases based on local clinical practice. Dose modifications followed the SmPC and were additionally informed by clinicians' experience, including the rate of platelet decline or rise.

The treatment goal was to maintain platelet counts within a safe, hemostatic range (approximately 50–150 × 10 ^9^ /L), although higher values were temporarily accepted in patients with unstable platelet fluctuations. Depending on disease activity, physicians could continue or taper concomitant treatments such as corticosteroids or immunosuppressants; IVIG was not used as maintenance but only as rescue therapy. Concomitant therapies were gradually reduced at the discretion of the treating physician, without a unified protocol.

Rescue therapy, defined as IVIG, platelet transfusion, or high-dose corticosteroids, was used only in cases of significant bleeding or a sudden decline in platelet counts, typically when platelet counts fell below 10 × 10 ^9^ /L or when clinically relevant bleeding symptoms were present. Twelve out of 142 patients (8.5%) required rescue therapy during the first month of avatrombopag treatment. The planned follow-up duration was 12 months, although patients could discontinue avatrombopag earlier or later, depending on clinical response. Discontinuation occurred due to TFR, AEs, lack of response (NR), or loss of response (LR). Slower dose tapering was permitted in selected patients due to transient platelet “peaks” followed by spontaneous decline on stable doses.

### Variable Definitions and Outcomes

Treatment response was evaluated according to International Working Group criteria. A complete response (CR) was defined as achieving a platelet count ≥ 100 × 10 ^9^ /L without significant bleeding or the need for rescue therapy. A response (R) required a platelet count ≥ 30 × 10 ^9^ /L and at least a twofold increase from baseline, also without clinically relevant bleeding. Patients who failed to meet these criteria were classified as nonresponders (NR). Responses were assessed at predefined time points (T1, T3, T6, and T12). Time to response was defined as the interval from avatrombopag initiation to the first protocol-defined assessment at which response criteria were fulfilled (earliest uniform assessment at approximately 30 days). A durable response was defined as maintaining CR or R for at least 6 months while on avatrombopag. In contrast, a TFR required sustaining CR or R for at least 12 months after discontinuation of all ITP therapies.

### Statistical Analysis

Results were expressed as mean with standard deviation (mean ± SD) for continuous variables with normal distribution (tested by the Shapiro–Wilk test) or as a median with interquartile range (median, 25–75%) in all other cases. Categorical variables are presented as percentages related to the size of the subgroups. The nonparametric Wilcoxon signed-rank test was applied for the comparison of paired data over time. Predictors of treatment response and of loss of response were evaluated by binary logistic regression. Missing data in the logistic regression analyses were not imputed; cases with missing values were excluded from the analysis. A value of *p* < 0.05 was considered statistically significant. All modeling was performed in StatsDirect statistical software (v4.0.4) and using the R program (v4.5.1). 31

### Ethical Considerations

The study protocol was submitted to the local bioethics committee, which confirmed that, owing to the retrospective observational design and the use of fully anonymized data, formal ethical approval and written informed consent were not required.

## Results

### Baseline Characteristics

A total of 142 patients were included. The median age at avatrombopag initiation was 54 years (IQR: 39–68). Nearly all had chronic ITP (92.3%), with 7.7% classified as persistent disease. The median time from ITP diagnosis to avatrombopag initiation was 6.0 years (IQR: 2.0–13.8), reflecting a long-standing and treatment-refractory population. Patients were heavily pretreated, having received a median of three prior treatment lines (IQR: 2–4). All patients had previously been treated with corticosteroids, while other common therapies included IVIG (61.3%), azathioprine (33.1%), cyclosporine (26.1%), danazol (14.8%), rituximab (13.4%), and MMF (14.8%). Prior exposure to TPO-RAs was documented in 36.6% of patients, most frequently eltrombopag, followed by romiplostim. Thirteen patients (9.2%) had undergone splenectomy, and thrombotic events had occurred in 12.0% ( Table 1 ).

**Table 1:** Baseline characteristics of patients at avatrombopag initiation ( *n* = 142)

Parameters	Value
Age, y	
Mean ± SD	53.0 ± 18.1
Median [IQR]	54 [39–68]
Sex	
Women	74 [52.1%]
Man	68 [47.9%]
ITP phase	
Newly diagnosed	0
Persistent	11 [7.7%]
Chronic	131 [92.3%]
Number of prior treatment lines	3 [2–4]
Previous therapies	
Corticosteroids	142 [100.0%]
IVIG	87 [61.3%]
Azathioprine	47 [33.1%]
Cyclosporine	37 [26.1%]
MMF (mycophenolate mofetil)	21 [14.8%]
Cyclophosphamide	2 [1.4%]
Danazol	21 [14.8%]
Rituximab	19 [13.4%]
TPO therapy	52 [36.6%]
TPO-RA (eltrombopag)	50 [35.2%]
TPO-RA (romiplostim)	11 [7.7%]
Fostamatinib	1 [0.7%]
Splenectomy	13 [9.2%]
Spleen size [cm]; *n* = 55	10.0 [10.0–12.0]
Thrombotic events	17 [12.0%]
Adverse events during prior treatment	11 [7.7%]
Rescue therapy	22 [15.5%]
Hospitalization	35 [24.6%]
Major bleeding events	8 [5.6%]
TPO switching	47 [33.1%]
Previous agent	Eltrombopag 40 [85.1%] Romiplostim 7 [14.9%]
Current concomitant treatment	70 [49.3%]
Current concomitant ITP therapy	59 [41.5%]
Corticosteroids	54
MMF	2
Azathioprine	10
Cyclosporine	8
Cyclophosphamide	0
Danazol	2
Anticoagulants	5
Antiviral drugs	2
Antidiabetic drugs	2
Antihypertensives	8
Levothyroxine	2
Statins	0
Bleeding score	0 [0–2.0]
Platelet count (PLT; 10 ^9^ /L)	21.5 [8.0–36.2]
Mean platelet volume (MPV; fl); *n* = 63	12.3 [11.4–13.0]
Platelet large cell ratio (PLCR; %); *n* = 54	44.3 [39.1–48.8]
Immature reticulocyte fraction (IRF; %); *n* = 10	11.1 [6.7–34.1]
Immature platelet fraction [%]; *n* = 82	19.5 [12.0–31.5]
Immature platelet fraction (IPF; 10 ^9^ /L); *n* = 75	5.1 [2.7–7.8]
Hemoglobin (Hgb; g/dL); *n* = 141	13.6 [12.0–14.8]
White blood cell count (WBC; 10 ^9^ /L); *n* = 140	7.5 [5.8–9.9]
Neutrophils [10 ^9^ /L]; *n* = 135	4.3 [3.1–6.9]
Lymphocytes [10 ^9^ /L]; *n* = 132	1.76 [1.30–2.47]
Monocytes [10 ^9^ /L]; *n* = 132	0.60 [0.49–0.82]
Eosinophils [10 ^9^ /L]; *n* = 131	0.10 [0.05–0.15]
Basophils [10 ^9^ /L]; *n* = 131	0.04 [0.02–0.06]
Alanine aminotransferase (ALT; IU/l); *n* = 131	23.0 [16.0–37.0]
Aspartate aminotransferase (AST; IU/l); *n* = 130	23.0 [18.0–33.0]
C-reactive protein (CRP; mg/L); *n* = 68	2.00 [0.97–5.00]
Bilirubin [mg/dL]; *n* = 123	0.57 [0.38–0.76]
Urea [mg/dL]; *n* = 70	36.1 [28.1–45.1]
Creatinine [mg/dL]; *n* = 110	0.80 [0.71–0.97]
MGUS	4 [2.8%]
Time from ITP diagnosis to avatrombopag initiation [y]	6.0 [2.0–13.8]

Abbreviations: ITP, immune thrombocytopenia; IVIG, intravenous immunoglobulin; MGUS, monoclonal gammopathy of undetermined significance; MMF, mycophenolate mofetil; *n* , number of patients; TPO-RA, thrombopoietin receptor agonist.

Note: Values are presented as mean ± standard deviation or median [interquartile range], unless otherwise indicated. Missing values indicate unavailable baseline laboratory data.

At the time of avatrombopag initiation, the median platelet count was 21.5 ×10 ^9^ /L (IQR: 8.0–36.2), and the median bleeding score was 0 (IQR: 0–2.0). Rescue therapy prior to treatment was required in 15.5% of patients, and 24.6% had been hospitalized. Nearly half of the cohort (49.3%) were receiving concomitant medications, most commonly corticosteroids, immunosuppressants, or other supportive therapies. Hemoglobin, leukocyte counts, and biochemical parameters were generally within expected ranges, although missing data were noted for several laboratory indices (including MPV, PLCR, IPF, IRF, CRP, and renal function markers; Table 1 ). The median follow-up duration for the entire cohort was 6 months (IQR: 3–12; range: 1–12 months).

### Platelet Response Over Time

Platelet responses to avatrombopag were rapid and substantial. At 1 month, among 138 patients with evaluable platelet counts, 66 (47.8%) achieved a CR and 42 (30.4%) a response (R), yielding an overall response rate of 78.3% (CR + R). At 3, 6, and 12 months, overall response rates among evaluable patients were 84.1% (CR: 58.4%, R: 25.7%; *n* = 113), 90.8% (CR: 60.9%, R: 29.9%; *n* = 87), and 96.8% (CR: 68.2%, R: 28.6%; *n* = 63), respectively, with only a small proportion of patients classified as nonresponders at each time point ( Table 2 ).

**Table 2:** Clinical response to avatrombopag over time, including dosing, and platelet count outcomes

	Baseline	1 mo	3 mo	6 mo	12 mo
Number of patients	*n* = 142	*n* = 138	*n* = 113	*n* = 87	*n* = 63
Mean weekly dose	NA	144.4 ± 72.1 140.0 [80.0–140.0]	135.4 ± 85.4 140.0 [60.0–200.0]	125.0 ± 80.5 140.0 [60.0–140.0]	129.1 ± 77.1 140.0 [60.0–180.0]
Response category					
CR	NA	66 [47.8%]	66 [58.4%]	53 [60.9%]	43 [68.2%]
R	NA	42 [30.4%]	29 [25.7%]	26 [29.9%]	18 [28.6%]
NR	NA	30 [21.7%]	18 [15.9%]	8 [9.2%]	2 [3.2%]
Platelet count outcomes					
PLT [10 ^9^ /L]	21.5 (8.0–36.2)	94.0 (49.5–165.5) *p* < 0.0001 a	114.0 (61.5–177.5) *p* < 0.0001 a	114.5 (79.0–164.2) *p* < 0.0001 a	124.0 (86.0–174.0) *p* < 0.0001 a

Abbreviations: CR, complete response; NA, not applicable; NR, no response; R, response.

Note: Values are presented as mean ± standard deviation or median (interquartile range).

a Versus baseline.

**Fig. 1:**
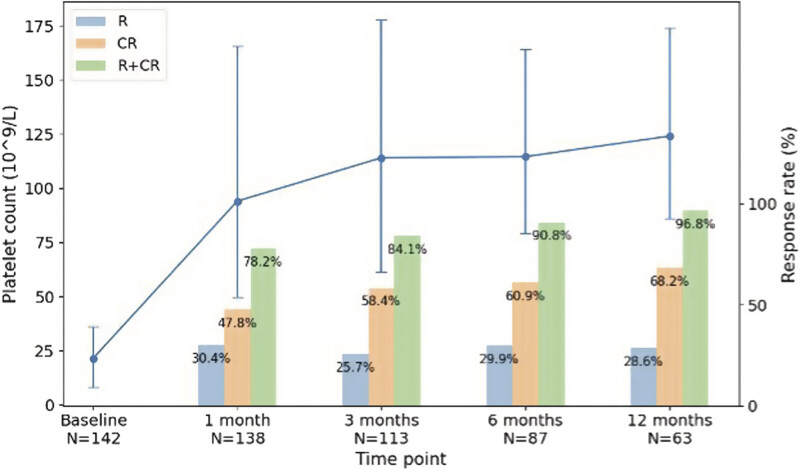
Platelet count dynamics during 12 months of avatrombopag treatment. Median platelet counts (with interquartile ranges) at baseline and during avatrombopag therapy at baseline, 1, 3, 6, and 12 months. The number of evaluable patients at each time point is shown beneath the *x* -axis. CR, complete response; R, response.

When all 142 enrolled patients were considered in an intention-to-treat framework, counting nonevaluable patients as nonresponders, overall response rates were 76.1% (108/142), 66.9% (95/142), 55.6% (79/142), and 43.0% (61/142) at 1, 3, 6, and 12 months, respectively. The number of evaluable patients at these time points was 138, 113, 87, and 63, respectively ( Fig. 1 ). Thus, the apparent decline in response rates over time in the full cohort primarily reflects treatment discontinuation and decreasing numbers of evaluable patients rather than a loss of efficacy among those who remained on therapy. Responses were observed early during treatment. The median time to first documented platelet response (R/CR) was 30 days, corresponding to the first scheduled assessment (T1). Because responses were evaluated at predefined intervals, the reported time reflects the timing of protocol-defined assessments rather than the exact biological onset of platelet increase. Similarly, the median time to R alone was 30 days (IQR: 30–30; range: 30–90; *n* = 48). Most CRs were also first documented at the initial evaluation (median: 30 days, IQR: 30–90), although a minority of patients achieved CR later during follow-up (maximum: 365 days; *n* = 97). When considering achievement of either R or CR, the median time remained 30 days (IQR: 30–30; range: 30–183; *n* = 121), indicating that platelet improvement was documented at the earliest uniform assessment in the majority of patients. Median platelet counts increased from 21.5 ×10 ^9^ /L (IQR: 8.0–36.2) at baseline to 94.0 ×10 ^9^ /L (49.5–165.5) at 1 month, and were maintained at 114.0 ×10 ^9^ /L (61.5–177.5), 114.5 ×10 ^9^ /L (79.0–164.2), and 124.0 ×10 ^9^ /L (86.0–174.0) at 3, 6, and 12 months, respectively ( *p* < 0.0001 vs. baseline for all time points; Table 2 ; Fig. 1 ). These data indicate sustained platelet recovery in patients continuing avatrombopag treatment over 12 months of follow-up.

### Durability of On-Treatment Response and Treatment-Free Response

Durable on-treatment responses were frequently observed among patients who remained on avatrombopag during extended follow-up. Notably, four patients (2.8%) achieved TFR after discontinuing therapy. The median time from avatrombopag initiation to the onset of TFR was 312 days (IQR: 119.5–567.5). At the time of treatment withdrawal, patients demonstrated robust platelet recovery, with a median platelet count of 183 ×10 ^3^ /µL (IQR: 167.0–200.5).

TFR was sustained for a median of 121.5 days (IQR: 86.5–252.0). Platelet counts remained stable throughout this period, with a median value of 201 ×10 ^3^ /µL (IQR: 168.0–205.0) at the end of observation. These findings indicate that a subset of patients can maintain adequate platelet levels without ongoing TPO-RA therapy, highlighting the potential for treatment de-escalation or discontinuation in selected responders (data not shown).

Among 121 patients who achieved response (R or CR), 79 (65.3%) maintained their response at the time of last follow-up, while 42 (34.7%) subsequently experienced loss of response. The time from achieving response to loss of response or last follow-up (herein referred to as relapse-free survival [RFS]) was analyzed for all responders. The median RFS among patients who lost response was 93 days (IQR: 60–153; range: 60–335). In the majority of these cases (38/42; 90.5%), loss of response was attributed to decreased treatment efficacy.

### Reasons for Avatrombopag Discontinuation and Subsequent Treatments

The overall duration of avatrombopag treatment was 91.0 days (IQR, 56.0–154.3; range, 26–658). The most common reasons for avatrombopag discontinuation were lack of response (NR; *n* = 10; 7%) and loss of response (LR; *n* = 8; 5.7%), followed by TFR ( *n* = 4; 2.8%). Other causes included platelet count fluctuations ( *n* = 2; 1.4%), AEs ( *n* = 3; 2.1%, bone pain, tongue candidiasis, hepatotoxicity), relocation abroad ( *n* = 1; 0.7%), and one death.

After stopping avatrombopag, patients transitioned to diverse therapies, most frequently romiplostim ( *n* = 4, 2.8%), corticosteroids ( *n* = 2; 1.4%), and eltrombopag ( *n* = 2; 1.4%). Additional treatments included splenectomy ( *n* = 2, 1.4%), cyclosporine ( *n* = 1, 0.7%), mycophenolate mofetil ( *n* = 1; 0.7%), azathioprine ( *n* = 2; 1.4%), azathioprine with steroids ( *n* = 1; 0.7%), and danazol with steroids ( *n* = 1; 0.7%; data not shown).

### Predictors of Treatment Response

In univariable logistic regression including 138 patients, several baseline factors were associated with the probability of achieving a platelet response (CR or R; Table 3 ). Higher hemoglobin concentration significantly increased the odds of response (OR: 1.47, 95% CI: 1.13–1.92, *p* = 0.004), whereas a higher bleeding score was associated with a reduced likelihood of response (OR: 0.83, 95% CI: 0.71–0.97, *p* = 0.019; Table 3 ). These associations may reflect underlying differences in disease burden at baseline; however, given the observational design and univariable analysis, no causal relationship can be inferred. Lower baseline urea levels were also associated with response (OR: 0.96, *p* = 0.027), although the substantial proportion of missing values limits interpretation. The strongest predictor of treatment response was the mean weekly avatrombopag dose during the first month. Lower dosing was strongly associated with achieving a response (OR: 0.756 per 10 mg/week, 95% CI: 0.681–0.840, *p* < 0.0001), reflecting standard clinical practice in which patients with more advanced or refractory disease typically require higher dose escalation to achieve platelet recovery, whereas patients with milder disease respond at lower doses.

**Table 3:** Baseline predictors of achieving response (R/CR) to avatrombopag ( *n* = 138)

Variable	OR	95% OR	Significance
Age [per decade]	1.068	0.806–1.413	NS ( *p* = 0.648)
Sex [female]	0.934	0.338–2.582	NS ( *p* = 0.895)
Number of prior treatment lines	0.918	0.678–1.242	NS ( *p* = 0.578)
Thrombotic events	1.061	0.221–5.108	NS ( *p* = 0.941)
Bleeding score	0.829	0.709–0.967	*p* = 0.019
Platelet count (PLT)	1.030	0.998–1.064	NS ( *p* = 0.069)
Mean platelet volume (MPV); *n* = 63	0.688	0.231–2.050	NS ( *p* = 0.502)
Platelet large cell ratio (PLCR); *n* = 54	0.948	0.804–1.119	NS ( *p* = 0.529)
Immature platelet fraction (%); *n* = 82	0.979	0.937–1.024	NS ( *p* = 0.361)
Immature platelet fraction (IPF); *n* = 75	0.962	0.898–1.032	NS ( *p* = 0.278)
Hemoglobin (Hgb); *n* = 141	1.474	1.131–1.922	*p* = 0.004
White blood cell count (WBC); *n* = 140	1.120	0.944–1.329	NS ( *p* = 0.192)
Neutrophils; *n* = 135	1.111	0.912–1.353	NS ( *p* = 0.296)
Lymphocytes; *n* = 132	1.348	0.732–2.483	NS ( *p* = 0.338)
Monocytes; *n* = 132	1.044	0.576–1.894	NS ( *p* = 0.886)
Eosinophils; *n* = 131	0.244	0.002–26.257	NS ( *p* = 0.555)
Basophils; *n* = 131	1.366	0.48–0.825	NS ( *p* = 0.962)
Alanine aminotransferase (ALT); *n* = 131	0.998	0.978–1.018	NS ( *p* = 0.816)
Aspartate aminotransferase (AST); *n* = 130	0.985	0.961–1.009	NS ( *p* = 0.221)
Bilirubin; *n* = 123	0.542	0.181–1.626	NS ( *p* = 0.274)
Urea; *n* = 70	0.956	0.919–0.995	*p* = 0.027
Creatinine; *n* = 110	0.992	0.935–1.053	NS ( *p* = 0.787)
Adverse events	0.330	0.078–1.392	NS ( *p* = 0.131)
Rescue therapy	0.392	0.123–1.255	NS ( *p* = 0.115)
Hospitalization	0.578	0.196–1.700	NS ( *p* = 0.319)
Major bleeding	0.391	0.072–2.117	NS ( *p* = 0.276)
Concomitant therapy	1.452	0.519–4.066	NS ( *p* = 0.477)
Mean weekly dose (month 1; 20 mg/d equivalents)	0.572	0.463–0.705	*p* < 0.0001

Abbreviations: CR, complete response; NS, not significant; R, response.

Note: Odds ratios (ORs) were calculated to identify baseline predictors of achieving a treatment response (R or CR) to avatrombopag. “Weekly dose” refers to the average avatrombopag dose administered during the first month of therapy. Missing values indicate unavailable baseline laboratory data.

No other baseline demographic, clinical, or biochemical parameter, including age, sex, treatment line, baseline platelet count, thrombotic history, liver function, leukocyte indices, or platelet production markers, was significantly associated with achieving a response. Interpretation of platelet indices (MPV, PLCR, IPF%, IPF) was limited by extensive missing data.

### Predictors of Loss of Response

Among 121 patients who initially achieved a platelet response (CR or R), none of the evaluated baseline variables predicted subsequent loss of response. Age, sex, line of therapy, bleeding score, baseline platelet count, thrombotic history, hematologic parameters, and biochemical indices all showed nonsignificant associations with the risk of losing response during follow-up (all *p* > 0.05; Supplementary Table S1 ). Similarly, early treatment characteristics, including the mean weekly avatrombopag dose at 1 month, did not predict later loss of response (OR: 1.001, *p* = 0.709). Platelet production indices (MPV, PLCR, IPF%, IPF) showed no predictive value, although interpretation was limited by substantial missingness.

These results indicate that the clinical determinants of initial treatment response differ from those underlying the durability of response, and that loss of response could not be predicted based on baseline characteristics or early treatment parameters in this cohort.

Consistent with the absence of identifiable predictors, the observed rates of loss of response declined over time. At 3 months, 54.8% of patients (23/42) experienced loss of response, compared with 38.9% (14/36) at 6 months and 30.0% (9/30) at 12 months. Among patients who lost response, the median time to loss was 90 days (IQR: 90–180; range: 85–365). The median weekly avatrombopag dose at 1 month was 80 mg (IQR: 80–140; range: 20–280).

### Safety

Avatrombopag was generally well tolerated during follow-up. Safety assessments were available for 138 patients at 1 month, 106 at 3 months, 84 at 6 months, and 62 at 12 months.

AEs were reported in 7/138 patients (5.1%) at 1 month, 7/106 (6.6%) at 3 months, 9/84 (10.7%) at 6 months, and 3/62 (4.8%) at 12 months. The apparent decrease in the absolute number of patients without reported AEs over time reflects the reduction in the number of patients remaining under active follow-up rather than an increase in event frequency.

Hepatotoxicity, defined as AST or ALT levels exceeding three times the upper limit of normal, was observed in only one patient during routine monthly liver function monitoring and occurred in the context of prior eltrombopag exposure. No thromboembolic events were observed during avatrombopag therapy in our cohort. Rescue therapy was required infrequently, and treatment discontinuation due to AEs was uncommon. Weekly blood counts and regular biochemical monitoring were performed during dose adjustments, which may have contributed to the favorable safety profile observed.

## Discussion

In this real-world analysis, we evaluated avatrombopag in adults with primary chronic ITP treated in routine practice in Poland. While randomized trials have established avatrombopag as an effective and generally well-tolerated second-line option, real-world data are crucial to understand its performance in heterogeneous, heavily pretreated populations. Our study contributes to this evidence base and represents, to our knowledge, the first real-world cohort from Poland.

In some patients, a 10 mg weekly avatrombopag regimen was used as an individualized dose adjustment based on platelet count trends and clinical judgement. This schedule is not part of the standard adult dosing algorithm described in the SmPC, which is based on 20 mg tablets with adjustment of dosing frequency. Although the 10 mg dose of avatrombopag has been evaluated in earlier dose-ranging clinical studies, 16 32 robust real-world evidence specifically supporting a 10 mg weekly regimen in adults is limited. Therefore, this approach should be considered a pragmatic, individualized modification used in selected cases rather than an evidence-based standard strategy.

Prior exposure to TPO-RAs was documented in 36.6% of patients, which appears lower than in some published real-world cohorts. For example, in the U.S.-based REAL-AVA 2.0 study, approximately 66% of patients had received a prior TPO-RA before initiating avatrombopag. 33 This difference likely reflects national reimbursement policies and treatment sequencing practices in Poland during the study period, where avatrombopag was not exclusively reserved for patients who had failed other TPO-RAs. The pivotal phase 3 trial demonstrated rapid and durable platelet responses with avatrombopag, with 65.6% responding by day 8 and one-third achieving durable responses. 26 Earlier phase 2 studies similarly showed dose-dependent platelet increases and sustained long-term efficacy. 16 Real-world observations extend these findings: in a large Chinese multicenter cohort, responses occurred in 83% and CRs in 62%, including patients previously unresponsive or intolerant to other TPO-RAs. 18

Within this context, our cohort showed comparably strong early activity. At 1 month, 76% achieved a platelet response, and 47% achieved CR. Among evaluable patients, platelet counts remained stably increased at 3, 6, and 12 months, consistently exceeding 94 ×10 ^9^ /L. Variations between our findings and those of clinical trials likely reflect differences in baseline platelet counts, chronicity, prior therapy intensity, and real-world treatment heterogeneity. Importantly, our findings confirm not only a high response rate but also a remarkably rapid onset of action, with a median time to R/CR of 30 days—the time of the first scheduled assessment. This highlights that avatrombopag provides early platelet stabilization in real-world practice, which is clinically valuable in patients with severe thrombocytopenia.

Reducing concomitant immunosuppression is an important therapeutic goal in chronic ITP. Clinical trial data showed that one-third of avatrombopag-treated patients were able to decrease background therapy, 26 with even higher rates reported in real-world cohorts. 18 In our study, nearly half of the patients were receiving concomitant ITP medications at treatment initiation; while the retrospective design did not allow systematic quantification of tapering or discontinuation, clinical documentation indicated that reduction of background therapy occurred in a subset of patients during avatrombopag treatment. TFR was observed in four patients (2.8%), indicating that durable disease control is achievable in selected individuals. Although this rate appears lower than the 10 to 30% sustained off-treatment responses reported in some clinical trial extensions and real-world studies of TPO-RAs, 34 35 such estimates vary widely depending on patient selection, treatment line, follow-up duration, and remission definitions.

Our cohort was heavily pretreated, with long-standing ITP (median disease duration: 6 years), factors associated with a lower likelihood of sustained remission. In addition, we applied a stringent definition of TFR requiring maintenance of response for at least 12 months after discontinuation of all ITP-directed therapies. These differences likely contributed to the lower observed TFR rate. Our analysis provides one of the few real-world evaluations of predictors of response to avatrombopag. Higher baseline hemoglobin and lower bleeding scores were associated with achieving a platelet response, indicating that patients with fewer bleeding manifestations and better physiological reserve respond more readily to TPO-RAs. Although direct evidence for these markers is limited, studies have shown that indicators of more advanced disease, particularly elevated endogenous thrombopoietin levels, predict reduced responsiveness to TPO-RAs, reflecting impaired marrow reserve. 36 The strongest predictor of response in our cohort was a lower mean weekly avatrombopag dose at 1 month, likely reflecting clinical practice patterns in which dose escalation is reserved for patients with insufficient early platelet recovery rather than a true inverse dose–response effect. The interpretation of platelet production markers (MPV, PLCR, IPF%, IPF) was limited by substantial missingness, preventing meaningful assessment of their prognostic value in this setting. While the number of prior treatment lines was evaluated as a predictor of response and was not significantly associated with treatment outcome (OR: 0.918, 95% CI: 0.678–1.242, *p* = 0.578), the specific impact of prior TPO-RA exposure was not separately analyzed and warrants further investigation.

By contrast, none of the baseline parameters, including age, sex, treatment history, platelet count, bleeding score, biochemical markers, or early avatrombopag dose, predicted loss of response among initial responders. This mirrors observations with other TPO-RAs, particularly romiplostim, where loss of response has been largely unpredictable and uncoupled from baseline clinical features. 17 Our findings highlight an ongoing challenge in ITP management: while predictors of initial response are emerging, determinants of response durability remain elusive.

Chronic ITP substantially affects daily functioning and quality of life. In a multinational survey, 89% of patients reported impairment in at least one domain, and many described treatment-related burdens, particularly the strict dietary restrictions required for eltrombopag. 4 A preference for TPO-RAs without food restrictions emerged clearly. Avatrombopag, administered once daily without dietary limitations, offers practical advantages in routine care. Although patient-reported outcomes were not formally assessed in our study, the favorable safety profile and sustained treatment exposure observed in our cohort may indirectly reflect good treatment acceptability. Prospective studies incorporating quality-of-life measures are warranted.

Safety is central to long-term ITP management. Although AEs were more frequent with avatrombopag than placebo in the phase 3 trial, exposure-adjusted rates were similar, and no new safety signals were observed. 26 A recent meta-analysis found no increased thrombotic risk with TPO-RAs, including avatrombopag, 28 and avatrombopag has not been associated with hepatotoxicity or bone marrow fibrosis. Data regarding the effects of avatrombopag on platelet function are limited but reassuring. In a randomized placebo-controlled study in patients with thrombocytopenia due to chronic liver disease, avatrombopag significantly increased platelet counts without enhancing in vivo platelet activation or in vitro platelet reactivity. 37 These findings suggest that avatrombopag-induced platelets are functionally competent but not hyperreactive. However, dedicated studies evaluating platelet function in patients with ITP remain scarce, and further research in this population is warranted. Our findings are consistent with the established safety profile of avatrombopag. AEs were infrequent, and thrombotic complications were rare and confined to patients with preexisting risk factors. Only one patient in the entire cohort developed severe hepatotoxicity requiring treatment discontinuation; notably, this individual had a prior history of eltrombopag-associated hepatotoxicity. Rescue therapy and discontinuation due to toxicity were otherwise uncommon. Nevertheless, given the retrospective design and sample size, larger multicenter registries will be important to further characterize long-term safety, particularly in high-risk subgroups.

This study has several limitations. Its retrospective, multicenter design may have resulted in incomplete documentation and limits generalizability. The timing of platelet assessments differed from that in randomized trials, complicating comparisons of response durability. A further limitation is the progressive reduction in the number of evaluable patients over time. Importantly, this reduction does not solely reflect true attrition; a proportion of patients had their treatment initiated at a time point that did not allow completion of the full 12-month observation period before the data cut-off date and therefore should not be considered dropouts. Nonetheless, treatment discontinuation and genuine loss to follow-up did occur and may have influenced the estimation of long-term response durability and safety outcomes, particularly at 6 and 12 months. Although intention-to-treat analyses were applied where appropriate, the decreasing sample size at later time points limits the precision of long-term estimates and may introduce attrition bias. Patient-reported outcomes were not collected. In addition, systematic genetic testing was not routinely performed; therefore, a subset of patients classified as having refractory primary ITP may have had underlying inborn errors of immunity or other immune-mediated thrombocytopenias. Finally, the study was not powered to detect rare AEs, underscoring the need for larger multicenter datasets.

## Conclusion

In this real-world Polish cohort of patients with ITP, avatrombopag achieved an overall platelet response rate of 78% at 1 month. Among evaluable patients, responses remained sustained over time (84.1% at 3 months, 90.8% at 6 months, and 96.8% at 12 months). Higher baseline hemoglobin levels and lower bleeding scores were associated with treatment response, whereas no clinical, laboratory, or early treatment parameters predicted loss of response. Loss of response occurred in 54.8% of patients at 3 months and decreased over time, to 38.9% at 6 months and 30.0% at 12 months. TFR was observed in 2.8% of patients. Among the 121 patients who achieved response (R or CR), 42 subsequently experienced loss of response, with a median relapse-free survival of 93 days (IQR: 60–153; range: 60–335). Overall, these findings support avatrombopag as an effective and well-tolerated option in routine ITP care.
