# Corrigendum: Efficacy and Safety of Avatrombopag Treatment in Immune Thrombocytopenia Patients in Poland: A Multicenter Study

**DOI:** 10.1055/a-2928-1127

**Published:** 2026-09-10

**Authors:** Michal Witkowski, Jagoda Rytel, Justyna Kozinska, Jolanta Oleksiuk, Jaroslaw Piszcz, Weronika Piszczek, Elzbieta Wiater, Jacek Kwiatkowski, Jaroslaw Dybko, Janusz Halka, Radoslaw Bogucki, Wiktoria Ryzewska, Tadeusz Robak

**Affiliations:** 1 Hematology Outpatient Clinic, Copernicus Memorial Hospital, Lodz, lodzkie, Poland; 2 Department of Science and Technology, Foundation for Leukemia Patients, Lodz, Poland; 3 37803 Department of Hematology, Transplantation and Internal Medicine, Medical University of Warsaw, Warsaw, Masovian Voivodeship, Poland; 4 Department of Hematooncology and Bone Marrow Transplantation, Medical University of Lublin, Lublin, Lublin Voivodeship, Poland; 5 Hematology Clinic, University Clinical Hospital in Bialystok, Bialystok, Podlaskie Voivodeship, Poland; 6 Department of Hematology, Rydygier Provincial Polyclinical Hospital, Torun, Poland; 7 Myeloma and Amyloidosis Treatment Center, Department of Hematology and Bone Marrow Transplantation, Nicolaus Copernicus Hospital, Torun, Poland; 8 Hematology and Transplantology Center, Lower Silesian Oncology, Pulmonology and Hematology Center, Wroclaw, Poland; 9 Department of Hematology and BMT, Clinical Hospital of the Ministry of Internal Affairs and Administration with the Warmia-Masuria Oncology Centre, Olsztyn, Poland; 10 Department of General Hematology and Internal Medicine, Copernicus Memorial Hospital, Lodz, Poland; 11 Haematology Clinic, Medical University of Lodz, Lodz, Lodz Voivodeship, Poland



## Notes

The authors have brought to the Publisher’s attention that there were changes in the list of authors in the byline, in the above article published in *TH Open* , Volume 10, 2026, page: a28683290 (DOI: 10.1055/a-2868-3290). The article is now corrected in the online version, and the author sequence in the byline has been corrected as above.

