# Supplementary material for: Efficacy and Safety of Avatrombopag Treatment in Immune Thrombocytopenia Patients in Poland: A Multicenter Study
Source: TH Open. 2026 May 13;10:a28683290. doi: 10.1055/a-2868-3290 (PMC13334203; doi:10.1055/a-2868-3290)
Supplement: Supplementary file 1 — Supplementary Material [file 10-1055-a-2868-3290_28762228.pdf]

Supplementary Table 1. Baseline predictors of loss of response among responders (N = 121)

| Variable | OR | 95% OR | Significance |
|---|---|---|---|
| **Age (per decade)** | 1.094 | 0.882-1.357 | NS (P=0.413) |
| **Sex (female)** | 1.377 | 0.632-2.998 | NS (P=0.421) |
| **Line of therapy** | 1.178 | 0.929-1.494 | NS (P=0.177) |
| **Thrombotic events** | 0.529 | 0.140-2.000 | NS (P=0.348) |
| **Bleeding score** | 0.938 | 0.779-1.130 | NS (P=0.502) |
| **Platelet count (PLT)** | 0.998 | 0.984-1.011 | NS (P=0.720) |
| **Mean platelet volume (MPV);** N = 64 | 1.411 | 0.915-2.176 | NS (P=0.119) |
| **Platelet large cell ratio (PLCR);** N = 72 | 0.982 | 0.936-1.031 | NS (P=0.461) |
| **Immature platelet fraction (%);** N = | 1.003 | 0.967-1.041 | NS (P=0.872) |
| **Immature platelet fraction (IPF);** N = | 0.918 | 0.794-1.061 | NS (P=0.245) |
| **Hemoglobin (Hgb);** N = | 0.911 | 0.739-1.123 | NS (P=0.383) |
| **White blood cell count (WBC);** N = | 0.919 | 0.825-1.025 | NS (P=0.130) |
| **Neutrophils;** N = | 0.875 | 0.757-1.010 | NS (P=0.069) |
| **Lymphocytes;** N = | 0.862 | 0.567-1.309 | NS (P=0.485) |
| **Monocytes;** N = | 0.596 | 0.164-2.169 | NS (P=0.432) |
| **Eosinophils;** N = | 0.805 | 0.019-34.854 | NS (P=0.910) |
| **Basophils;** N = | 0.001 | 0-10.654 | NS (P=0.099) |
| **Alanine aminotransferase (ALT);** N = | 1.001 | 0.986-1.017 | NS (P=0.854) |
| **Aspartate aminotransferase (AST);** N = | 1.001 | 0.979-1.025 | NS (P=0.901) |
| **Bilirubin;** N = | 0.765 | 0.237-2.473 | NS (P=0.655) |
| **Urea;** N = 70 | 1.021 | 0.975-1.069 | NS (P=0.378) |

| | | | |
|---|---|---|---|
| **Creatinine;** N = | 0.959 | 0.789-1.165 | NS (P=0.674) |
| **Adverse events** | 1.394 | 0.315-6.166 | NS (P=0.661) |
| **Rescue therapy** | 1.727 | 0.601-4.959 | NS (P=0.310) |
| **Hospitalization** | 0.828 | 0.328-2.088 | NS (P=0.689) |
| **Major bleeding** | 0 | 0 | NS (P>0.999) |
| **Concomitant treatment** | 1.444 | 0.663-3.145 | NS (P=0.355) |
| **Mean weekly dose (1 month)** | 1.001 | 0.995-1.008 | NS (P=0.709) |

Odds ratios (ORs) were calculated to identify baseline predictors of subsequent loss of response among patients who initially achieved a platelet response (R or CR) to avatrombopag. “Weekly dose” refers to the average avatrombopag dose administered during the first month of therapy. Missing values indicate unavailable baseline laboratory data.
